# *CXCL10* potentiates immune checkpoint blockade therapy in homologous recombination-deficient tumors

**DOI:** 10.7150/thno.59056

**Published:** 2021-05-24

**Authors:** Zhiwen Shi, Jianfeng Shen, Junjun Qiu, Qingguo Zhao, Keqin Hua, Hongyan Wang

**Affiliations:** 1 Obstetrics and Gynecology Hospital, NHC Key Laboratory of Reproduction Regualtion, Shanghai Institute of Planned Parenthood Research, State Key Laboratory of Genetic Engineering at School of Life Sciences, Institute of Reproduction and Development, Fudan University, Shanghai 200032, China.; 2 Shanghai Key Laboratory of Female Reproductive Endocrine Related Diseases, Institute of Metabolism and Integrative Biology, Institutes of Biomedical Sciences, Fudan University, Shanghai 200433, China.; 3 Department of Ophthalmology, Ninth People's Hospital, Shanghai JiaoTong University School of Medicine, Shanghai, 200025, China.; 4 Shanghai Key Laboratory of Orbital Diseases and Ocular Oncology, Shanghai, 200025, China.; 5 Children's Hospital of Fudan University, Shanghai 201100, China.

**Keywords:** HRD, cGAS-STING, TIME, *CXCL10*, immunotherapy

## Abstract

**Background:** Homologous recombination deficiency (HRD) is a common molecular characteristic of genomic instability, and has been proven to be a biomarker for target therapy. However, until now, no research has explored the changes in the transcriptomics landscape of HRD tumors.

**Methods:** The HRD score was established from SNP array data of breast cancer patients from the cancer genome atlas (TCGA) database. The transcriptome data of patients with different HRD scores were analyzed to identify biomarkers associated with HRD. The candidate biomarkers were validated in the gene expression omnibus (GEO) database and immunotherapy cohorts.

**Results:** Based on data from the gene expression profile and clinical characteristics from 1310 breast cancer patients, including TCGA database and GEO database, we found that downstream targets of the cGAS-STING pathway, such as *CXCL10,* were upregulated in HRD tumors and could be used as a predictor of survival outcome in triple-negative breast cancer (TNBC) patients. Further comprehensive analysis of the tumor immune microenvironment (TIME) revealed that the expression of *CXCL10* was positively correlated with neoantigen load and infiltrating immune cells. Finally, *in vivo* experimental data and clinical trial data confirmed that the expression of *CXCL10* could be used as a biomarker for anti-PD-1/PD-L1 therapy.

**Conclusions:** Together, our study not only revealed that *CXCL10* is associated with HRD but also introduced a potential new perspective for identifying prognostic biomarkers of immunotherapy.

## Introduction

Immune checkpoint blockade (ICB) therapy produces an average objective response rate of up to 50% in various types of solid tumors with DNA deficient mismatch repair 1,2. This is an important finding, indicating that ICB inhibitors can be used as “broad-spectrum drugs” for tumor treatment. Generally, dMMR tumors are characterized by an increased number of mutations and enhanced T-cell infiltration; however, a considerable number of dMMR tumors have a high mutation burden but lack sufficient T-cell infiltration and respond poorly to ICB therapy 3. Usually, the response to ICB therapy depends on the following biomarkers: 1) the number of neoantigens derived from genome instability, such as the tumor mutation burden (TMB) and dMMR, and 2) tumor-infiltrating lymphocytes (TILs), mainly CD4^+^ T cells, CD8^+^ T cells and gene signals that interfere with the activity of T cells, such as those affecting PD-1/PD-L1/CTLA4 function 4,5. The effective activation of T cells not only depends on neoantigens but also requires costimulatory molecular signals recognized by the innate immune system. As an important part of innate immune recognition, cytoplasmic DNA recognition mediated by the cGAS-STING pathway was reported in 2013 as a breakthrough finding 6.

In the recent issue of *Cancer Cell*, Lu and Guan et al. demonstrated that the activation of the cGAS-STING pathway in tumor tissues was significantly and positively correlated with the prognosis of patients bearing dMMR tumors but not that of patients with pMMR (proficient MMR) tumors. The results of their *in vitro* experiments showed that antigen-presenting cells nurtured by dMMR but not pMMR tumors could strongly promote T-cell proliferation, whereas this phenomenon was abolished when STING was ablated in dMMR tumor cells 7.

In addition to dMMR, homologous recombination deficiency (HRD) also induces genomic instability and serves as an effective therapeutic biomarker for breast cancer and ovarian cancer 8-11.

To explore whether there is a possible connection between the ICB therapeutic biomarkers in genomically unstable tumors elicited by HRD, we extracted the HRD score and transcriptome data from the Cancer Genome Atlas breast cancer cohort (TCGA-BRCA). By analyzing the transcriptome data of patients with HRD tumors, we found that downstream targets of the cGAS-STING pathway, such as *CXCL10*, were positively associated with HRD. Furthermore, we discovered a positive relationship between the* CXCL10* expression and tumor immune microenvironment (TIME), including infiltrating immune cells, neoantigen load and immune checkpoint blockade (ICB). Moreover, high *CXCL10* expression was able to be used as biomarkers for anti-PD-1/PD-L1 therapy, and the predictive effect of *CXCL10* was better than that of PD-1/PD-L1. Our research perspectives and methods provide a possible direction for immunotherapy. The results of this study may be valuable for understanding the relationship between the genomic instability and TIME and improving the clinical outcome of patients receiving anti-PD-1/PD-L1 therapy.

## Materials and Methods

### Data collection and processing

Patients' RNA sequencing data, SNP array data and corresponding clinical follow-up information were downloaded from the publicly available the Cancer Genome Atlas (TCGA) database (https://portal.gdc.cancer.gov) and the NCBI Gene Expression Omnibus (GEO) database 12. RNA sequencing data were normalized as transcripts per million (TPM) by using the R. SNP array data were processed using Affymetrix Power Tools and PennCNV. The somatic mutation counts, copy number variation (CNV), fraction genome altered scores (FGA: percentage of copy number altered chromosome regions out of measured regions) and MSIsensor score (microsatellite instability detection using paired tumor-normal sequence data) were obtained from the cBioPortal database (http://www.cbioportal.org/study?id = brca_tcga_pan_can_atlas_2018). In total, 1055 TCGA-BRCA samples data were extracted; 255 GEO samples data were extracted (E-MTAB-365, GSE19615, GSE21653, GSE2603 and GSE31519) (http://kmplot.com/analysis/index.php?p = service&cancer = breast ). Please refer to Table S1 for the clinical information of patients included in this study. Single-cell RNA-seq of Triple-negative breast cancer (TNBC) patients were obtained from GSE118389 13. The transcriptome profile and clinical information from immunotherapy cohorts were obtained from Imvigor210 14,15. The RNA-seq data of immune checkpoint treated tumors from TNBC murine models were downloaded from GSE124821 16.

### HRD score analysis

Loss of heterozygosity (LOH) was defined as the number of counts of chromosomal LOH regions shorter than whole chromosome and longer than 15 Mb 17. Large-scale State Transitions (LST) were defined as chromosome breakpoint (change in copy number or allelic content ) between adjacent regions each of at least 10 megabases obtained after smoothing and filtering shorter than 3 Mb small-scale copy number variation 18. Telomeric Allelic Imbalance (TAI) was defined as the number of regions with allelic imbalance which extend to the sub-telomere but do not cross the centromere 19. The HRD score was defined as the sum of TAI, LST, and LOH scores 20-22. The HRD score of each patient was shown in Table S2.

### Neoantigen load

The 4-digit HLA type for each sample was inferred using POLYSOLVER 23. Neo-epitopes were predicted for each patient by defining all novel amino acid 9mers and 10mers resulting from mutation in expressed genes (median >10 TPM in the tumor type) and determining whether the predicted binding affinity to the patient's germline HLA alleles was < 500 nM using NetMHCpan 24-26. The Neoantigen load of each patient was shown in Table S3.

### KEGG and Gene Ontology enrichment analysis

RNA-seq data (raw counts) analysis was conducted using the “edgeR” package of R 27 . Fold change > 1.5, adj. *p* < 0.05, TPM > 1 and genes with the first 75% of median absolute deviation (MAD) were set as the cutoffs to screen for differentially expressed genes (DEGs). Functional enrichment analysis of DEGs was performed by DAVID 28 to identify GO categories by their biological processes (BP), molecular functions (MF), or KEGG pathways.

### Identification of prognostic DEGs positively associated with the HRD score

Kaplan-Meier plots were generated to illustrate the relationship between patients' overall survival and gene expression levels of DEGs. The relationship was tested by log-rank test.

### Immune cells infiltration in bulk tumor gene expression data

In order to study the enrichment of immune cells, we used TIMER 29, an efficient algorithm for predicting immune cell infiltration of bulk tumor gene expression data (https://cistrome.shinyapps.io/timer/). For each sample, TIMER quantified the relative abundance of six types of infiltrating immune cells, including T cells, B cells, macrophages, neutrophiles and dendritic cells.

## Results

### The HRD Score reflects patients' genomic instability and can be used as a prognostic marker in patients with TNBC

According to the HRD-algorithm, LOH, TAI and LST were used as the basis for calculating the HRD score (Table S2). To explore the correlation between the HRD score and other hallmarks of genomic instability, including somatic mutation counts, fraction genome altered and microsatellite instability (MSI), the breast cancer patients were sorted in ascending order of HRD scores; and the bottom 20% and the top 20% of the patients were selected. As shown in Figure 2, the hallmarks of genomic instability were significantly higher in the top 20% HRD-score group than the bottom 20% HRD-score group (Wilcoxon signed-rank test, *P* < 0.0001, Figure 2A-C). However, the HRD score was not a good prognostic marker in the whole breast cancer cohort (Figure S1A). This result might be due to the endocrine therapy of breast cancer. Therefore, we analyzed the prognosis of triple-negative breast cancer (TNBC) patients by the HRD score. In the TNBC cohort, the Kaplan-Meier survival curve (Figure 2D) showed that overall survival of patients in the HRD-positive group (HRD scores > 26) was much longer than the cases in the HRD-negative group (HRD scores were ≤ 26) (log-rank test, *P* < 0.0001). Receiver operating characteristic (ROC) analysis showed that the HRD score had a good predictive effect on the prognosis of TNBC (Figure 2E).

### Downstream targets of the cGAS-STING pathway are associated with HRD

**Figure 1:**
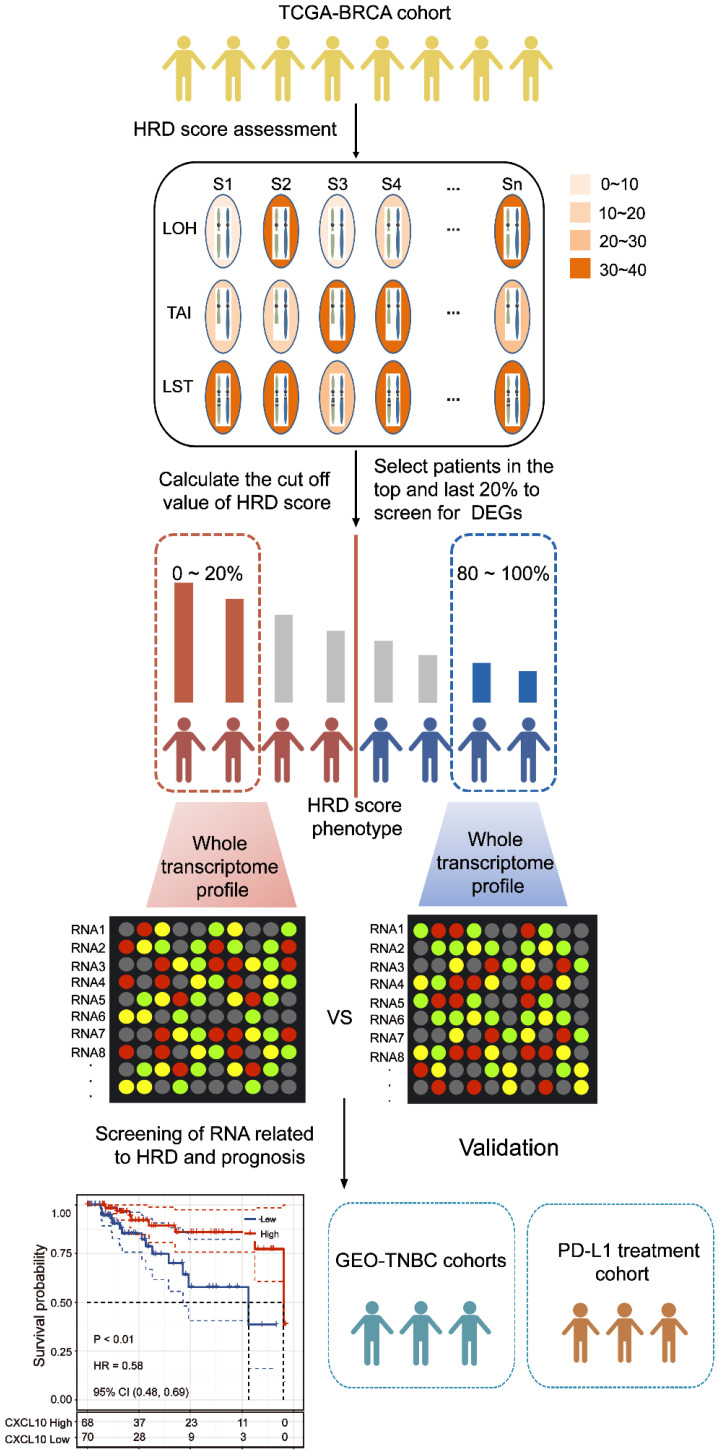
** Computational overview of HRD-related RNAs detection.** The columns reflected TCGA-BRCA samples, and the rows reflected three biomarkers of the HRD score. The color reflects the scores for each biomarker on each sample. HRD-related RNAs were detected by comparing the RNA expression profile between the top 20% patients with high HRD scores and the bottom 20% patients with low HRD scores.

**Figure 2:**
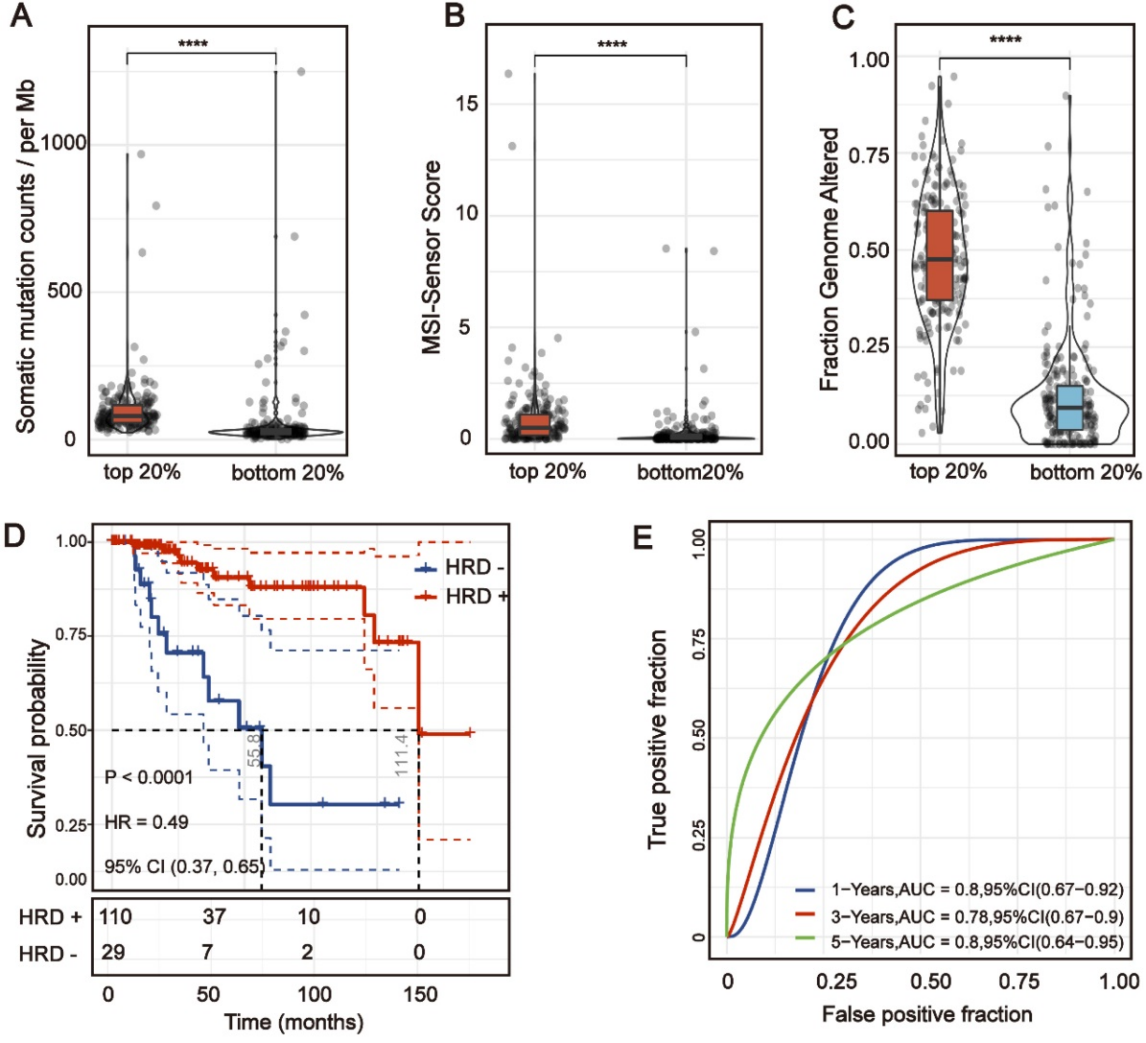
** The HRD Score reflects patients' genomic instability and can be used as a prognostic marker in patients with TNBC.** (A) Violin plot of somatic mutations in the top 20% HRD-score group and the bottom 20% HRD-score group. Somatic mutation counts in the top 20% HRD-score group were significantly higher than those in the bottom 20% HRD-score group (Wilcoxon signed-rank test, **** *P* < 0.0001). (B) Violin plot of MSI in the top 20% HRD-score group and the bottom 20% HRD-score group (Wilcoxon signed-rank test, **** *P* < 0.0001). (C) Violin plot of fraction genome altered in the top 20% HRD-score group and the bottom 20% HRD-score group (Wilcoxon signed-rank test, **** *P* < 0.0001). (D) Kaplan-Meier estimates of overall survival of patients with the HRD-positive or HRD-negative tumors calculated by the HRD score in the TCGA-TNBC cohort. (E) ROC curves analysis of the HRD score in the TCGA-TNBC cohort.

**Figure 3:**
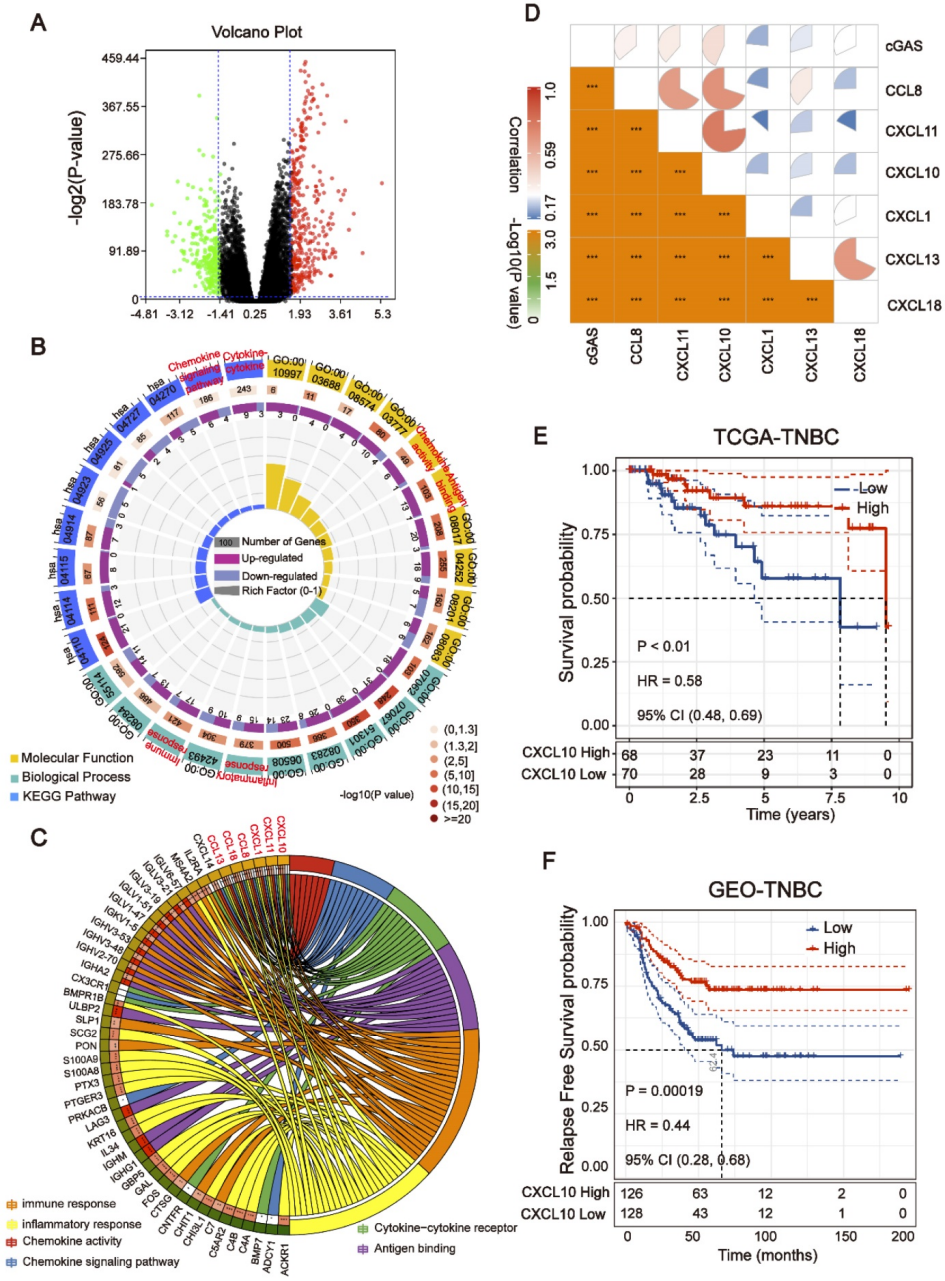
** Downstream targets of the cGAS-STING pathway are associated with HRD**. (A) Volcano plot of DEGs in the top 20% HRD-score group and the bottom 20% HRD-score group. The horizontal line at false discovery rate (FDR) < 0.05; vertical line at |log2FC| = 1.5; TPM > 1; MAD > 75%. (B) KEGG and Gene Ontology enrichment analysis of DEGs. The outermost ring represented the name of signaling pathways. The second outer ring represented the number of genes in signaling pathways, the heights of the columns in the inner ring indicate the proportion of DEGs in the total number of genes in the signaling pathway and the color depth represented the number of differential genes. (C) Chord plot depicting the relationship between genes and immune-related signaling pathways. The genes marked in red fonts refer to the most frequently repeated genes in immune-related signaling pathways. (D) Correlation between the expression of cGAS and the most frequently repeated DEGs in TCGA-BRCA cohort. The size and color of the pie chart represent the correlation coefficient. (E and F) Curve for overall survival is shown for high and low *CXCL10* expression in the TCGA-TNBC cohort and GEO-TNBC cohort.

To explore the transcriptomic signatures associated with HRD, the breast cancer patients were sorted in ascending order of HRD scores; and the bottom 20% and the top 20% of the patients were selected. We compared the difference of whole transcriptome between the top 20% HRD-score group and the bottom 20% HRD-score group (Figure 1). Utilizing the egdeR method, a total of 632 differentially expressed genes (DEGs) were screened out in breast cancer (Figure 3A). In breast cancer, the KEGG and GO cluster plots revealed that the up-regulated DEGs were enriched in immune-related signaling pathways such as immune response, chemokine signaling pathway and cytokine-cytokine receptor interaction in the top 20% HRD-score group (Figure 3B). Intriguingly, six upregulated genes (*CXCL1*,* CXCL10*,* CXCL11*, *CCL8*, *CCL13* and *CCL18*) among the immune-related signaling pathways appear repeatedly (Figure 3C). Notably, the six upregulated genes were closely correlated with the transcriptional data, indicating cGAS-STING signaling activation in the TCGA-BRCA cohort, as *CXCL10/11* had been reported to be downstream targets of the cGAS-STING pathway (Figure 3D, Pearson correlation coefficient, *R* > 0.4, *P* ≈ 0). The results from the Kaplan-Meier analysis showed that TCGA-TNBC patients with high expression of these six genes had prolonged survival, and *CXCL10* expression had the strongest predictive power for survival (Figure 3E and Figure S1B-F). The prognostic value of *CXCL10* was further verified in the GEO-TNBC cohort (Figure 3F). To confirm that up-regulated *CXCL10* expression is derived from HRD tumor cells, we re-analyzed single-cell RNA-seq data of TNBC patients 13. The box plot showed that the expression of *CXCL10* in the epithelial cells of the patient with *BRCA1* loss-of-function mutations was significantly higher than the expression of *CXCL10* in the epithelial cells of the HR proficient patient (Wilcoxon signed-rank test, **** *P* < 0.0001, Figure S2C).

### The *CXCL10* expression signature is positively associated with the tumor immune microenvironment

**Figure 4:**
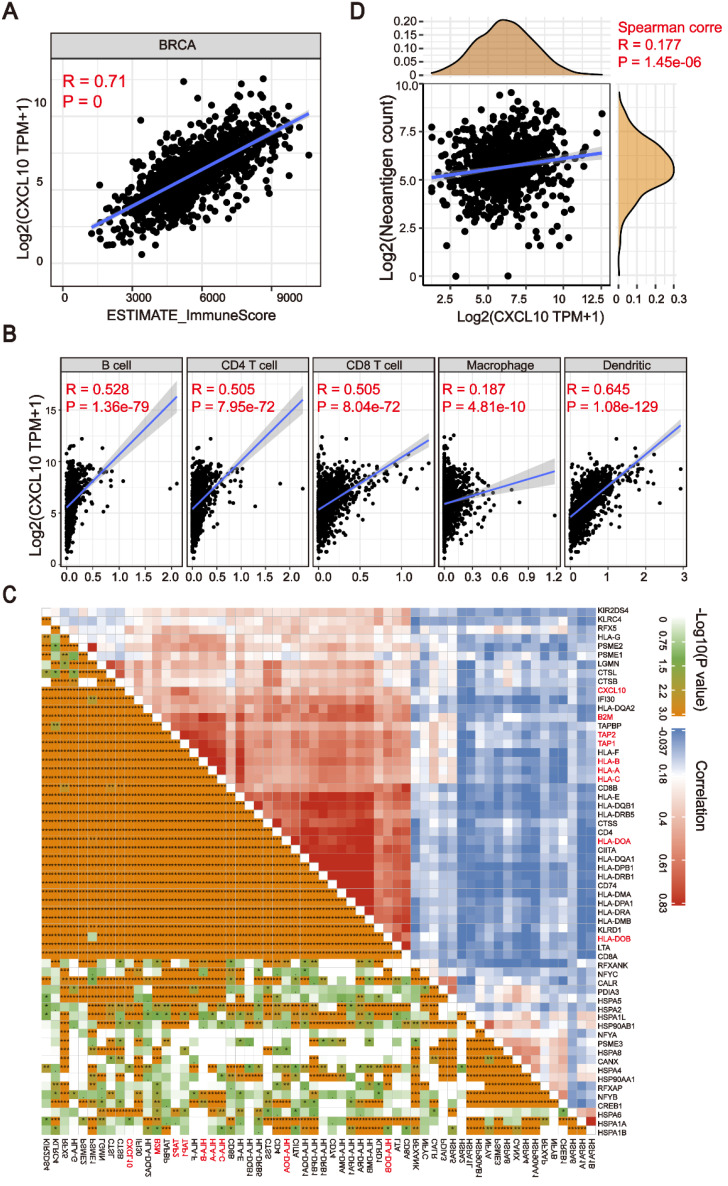
** The *CXCL10* expression signature is associated with TIME.** (A) Positive correlation between the *CXCL10* expression signature and ImmuneScore in the TCGA-BRCA cohort (Spearman's rank correlation coefficient, *R* = 0.71, *P ≈* 0). (B) Positive correlation between the *CXCL10* expression signature and immune cell subpopulations in the TCGA-BRCA cohort (Spearman's rank correlation coefficient, *P* < 0.0001). (C) Positive correlation between the *CXCL10* expression signature and antigen-related genes in the TCGA-BRCA cohort. (D) Positive correlation between the *CXCL10* expression signature and neoantigen load in the TCGA-BRCA cohort (Spearman's rank correlation coefficient, *P* < 0.0001).

The expression of Cytokines/Chemokines is essential for attracting immune cells 30,31. In order to explore the relationship between the chemokine signatures and tumor infiltrating immune cells, we first used the ESTIMATE algorithm to calculate the correlation between downstream targets of the cGAS-STING pathway and immune scores 32. As shown in Figure 4A, the expression of *CXCL10* was positively correlated with immune scores in breast cancer patients (Spearman's rank correlation coefficient, *R* > 0.6, *P ≈* 0). More importantly, we found that this correlation existed not only in the TCGA-BRCA cohort, but also in other cancer types (Figure S3). To further clarify the subtype of tumor infiltrating cells, the TIMER algorithm 33 was applied to estimate the association of various immune cell types with the *CXCL10* expression signature. As depicted in the scatter plot, the *CXCL10* upregulation correlated to the infiltration of dendritic cells and anti-tumor lymphocyte subpopulations (Spearman's rank correlation coefficient, *P* < 0.0001) (Figure 4B). Dendritic cells play a crucial role in antigen processing and presentation pathways, hinting that the expression of antigen-related genes might be associated with the expression of* CXCL10*. To prove this assumption, we explored the correlation of antigen-related genes with the *CXCL10* expression signature by using the Pearson correlation coefficient. We found that the expression of MHC class I/II (I: HLA-A, HLA-B, and HLA-C; II: HLA-DP, HLA-DM, HLA-DOA, HLA-DOB, HLA-DQ, and HLA-DR) and the key antigen binding (B2M, TAP1/2 and so on) molecules were positively correlated with the *CXCL10* expression signature (Spearman's rank correlation coefficient, *P* < 0.0001) (Figure 4C). We further identified that the predicted neoantigen load was positively correlated with the *CXCL10* expression signature (Spearman's rank correlation coefficient, *P* < 0.0001) (Figure 4D).

### The *CXCL10* expression signature is positively associated with ICB-related genes

**Figure 5:**
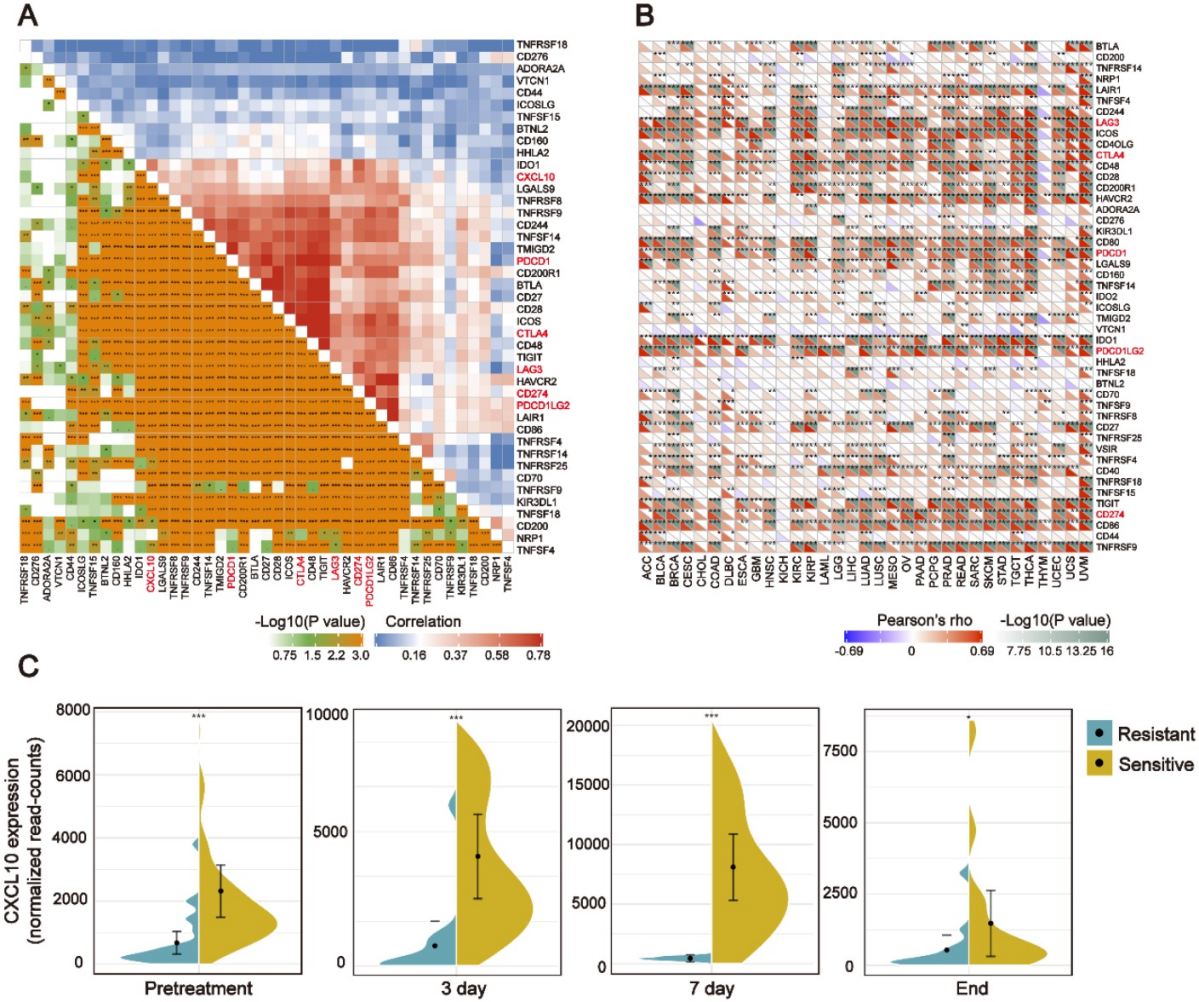
** The *CXCL10* expression signature is positively associated with ICB-related genes** (A) Correlation between the *CXCL10* expression signature and ICB-related genes in the TCGA-BRCA cohort. (B) Correlation between the *CXCL10* expression signature and ICB-related genes in the TCGA-pan cancer cohorts. (C) Violin plot depicting the expression of *CXCL10* in sensitive or resistant tumor tissues. Immunotherapy: anti-PD1 and anti-CTLA-4 combination therapy. From left to right: Before receiving immunotherapy, on the 3rd day of receiving immunotherapy, on the 7th day of receiving immunotherapy, and at the time point of the end of immunotherapy (Wilcoxon signed-rank test, ** *P* < 0.01, *** *P* < 0.001).

In recent years, ICB therapy, represented by anti-PD-1/L1, has played an increasingly important role in anti-tumor treatment 34. The characteristics of TIME and ICB-related genes have a profound impact on ICB therapy. Therefore, we collected more than 40 common ICB-related genes and analyzed the relationship between the* CXCL10* expression signature and ICB-related genes 35. As displayed by heatmap, the *CXCL10* expression was positively correlated with the expression of multiple ICB-related genes in the TCGA-BRCA cohort (Figure 5A). Ten of the most relevant ICB-related genes were: *LAG3*, I*COS, CTLA4, CD48, HAVCR2, PDCD1*(PD-1)*, PDCDILG2*(PD-L2)*, TIGIT, CD274*(PD-L1) and *CD86*. Generally, the key regulatory factors involved in immunity perform similar functions in different tissues. We thus explored the *CXCL10* expression signature and ICB-related genes across the cancer types. We found that the positive relationship between *CXCL10* and the ICB-related genes was not only present in breast cancer, but also in 32 other cancer types (Figure 5B).

### The *CXCL10* expression signature could be used as a potential biomarker for ICB therapy

**Figure 6:**
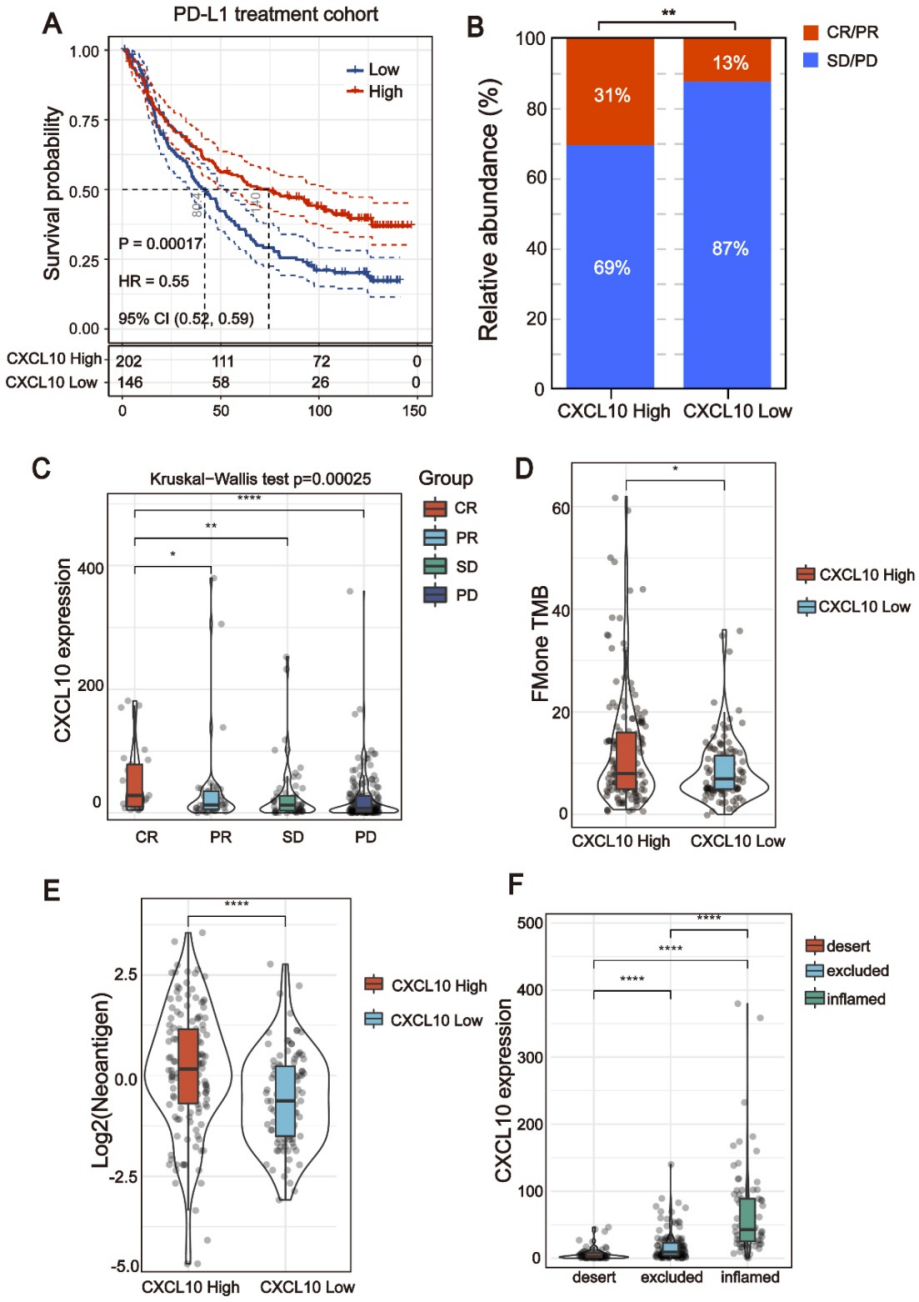
** The *CXCL10* expression signature could be used as potential biomarkers for ICB therapy.** (A) Curve for overall survival is shown for high and low *CXCL10* expression in the PD-L1 treatment cohort. (B and C) The proportion of immune response to anti-PD-L1 treatment in high versus low *CXCL10* expression subgroups. CR, complete response; PR, partial response; SD, stable disease; PD, progressive disease. (D and E) TMB and neoantigen load in the immunotherapy cohort were compared among distinct *CXCL10* expression signature subgroups (Wilcoxon signed-rank test, * P < 0.05, **** *P* < 0.0001). (F) The *CXCL10* expression signature in different immune phenotype subgroups. The tumor immunophenotype was defined according to immunohistochemistry results of the CD8 antibody (Wilcoxon signed-rank test, **** *P* < 0.0001) 14.

**Figure 7:**
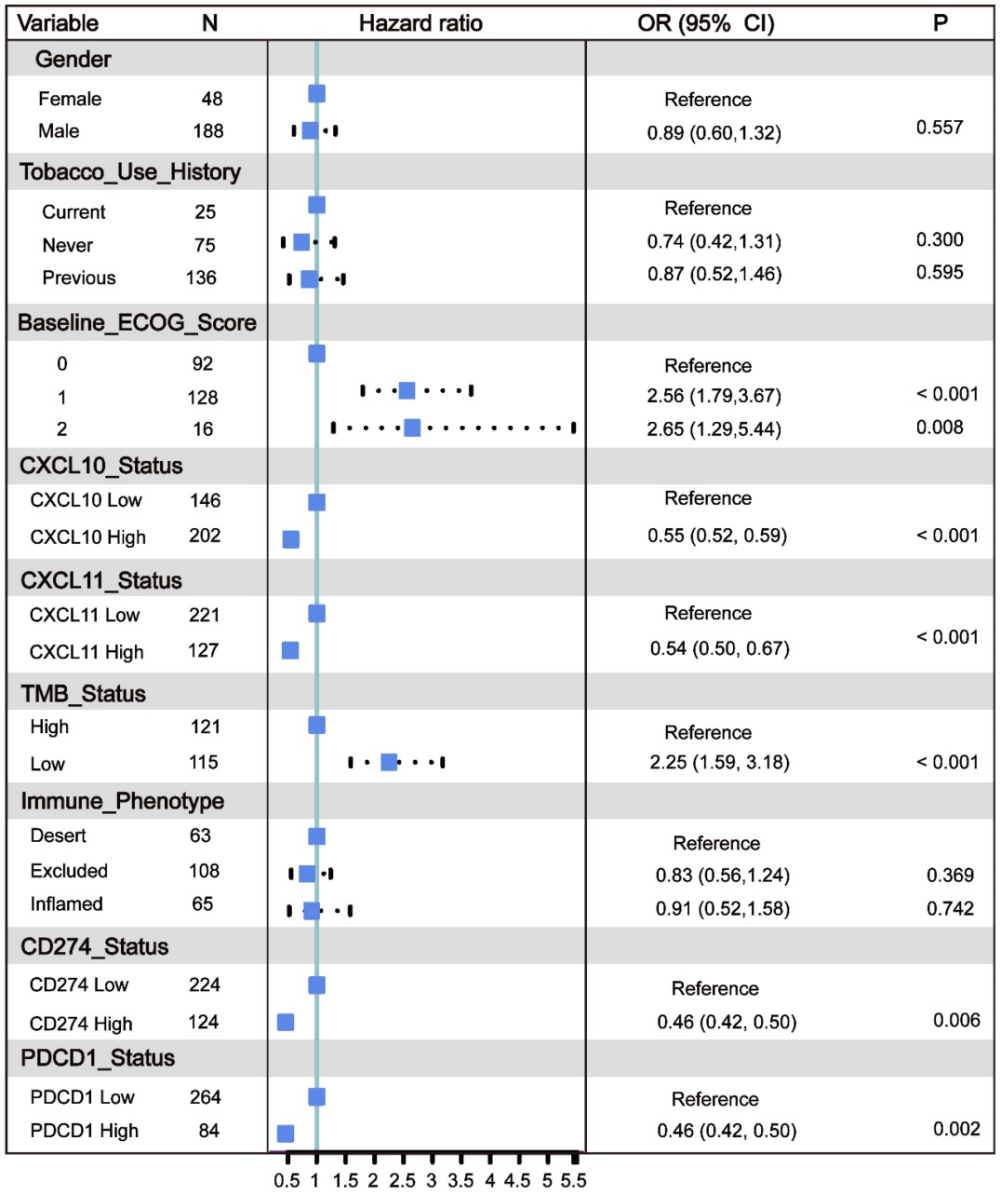
** Multivariate Cox regression analysis of the *CXCL10* expression signature with gender, smoking, ECOG score and immunophenotype were taken into account.** ECOG: Eastern Cooperative Oncology Group. TMB: Tumor Mutation Burden. Immune phenotype: Desert, CD8+ T cells are absent from the tumor and its periphery; Excluded, CD8+ T cells accumulated but do not efficiently infiltrate; Inflamed, CD8+ T cells infiltrate but their effects are inhibited.

All of the above results indicate that the* CXCL10* expression signature is closely related to the biomarkers for ICB therapy. Therefore, the RNA-seq data of immune checkpoint treated tumors from TNBC murine models 16 were used to investigate the role of *CXCL10* in ICB Therapy. As shown in Figure 5C, the expression of *CXCL10* in sensitive tumor tissues is significantly up-regulated at different time points of treatment compared with resistant tumor tissues (Wilcoxon signed-rank test, *** *P* < 0.001). To further confirm the predictive effect of *CXCL10* on ICB treatment, we collected the transcriptome profile and clinical information from an immunotherapy cohort (Imvigor210) of urothelial cancer (UC) treated with atezolizumab 14. In this cohort, tumor patients with high *CXCL10* expression exhibited markedly improved clinical benefits and significantly prolonged survival (Figure 6A). Significant therapeutic advantages and immune responses to PD-L1 blockades were observed in samples with high expression of *CXCL10* compared to those with low expression (Fisher extract test, *P* < 0.01, Figure 6B; Kruskal-Wallis H test, *P* < 0.001, Figure 6C). Further analysis revealed that TMB, neoantigen load and tumor infiltrating immune phenotype were significantly elevated in tumors with high expression of *CXCL10*, which was closely linked to immunotherapeutic efficacy (Figures 6D-F). Besides, the association between the expression of *CXCL10* and immunotherapy survival remained statistically significant after taking into account gender, smoking, ECOG score, immunophenotype and, PD-1/PD-L1 status (Figure 7).

## Discussion

**Figure 8:**
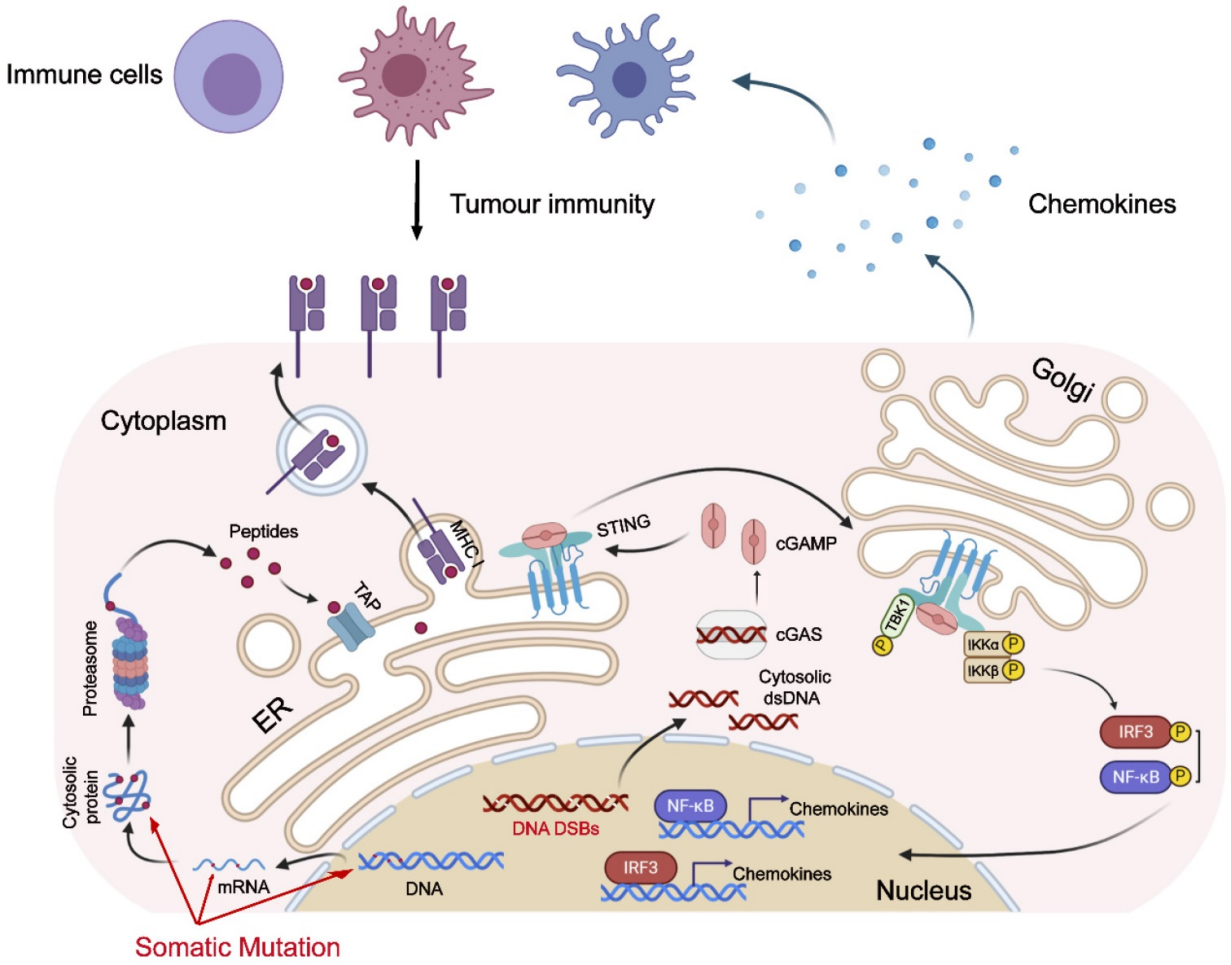
**Model for genomic instability-induced cGAS activation and immune response.** Genomic instability, caused by dMMR or HRD, leads to tumor cells bearing a higher neoantigen load. Additionally, the instability of the tumor genome also causes the activation of the cGAS-STING pathway, which in turn up-regulates the expression of chemokines and attracts immune cells to migrate to tumor tissue.

Emerging evidence has shown the importance of the cGAS-STING pathway in tumor immunotherapy 36,37. Recent studies have found that the cGAS-STING pathway being activated in the setting of genome instability can be attributed to dMMR 38, PARP inhibitor treatment, or functional loss of *BRCA1/2* genes 39, and it predicts a better prognosis of tumor patients. While these previous studies, combined with our research, fully prove that genomic instability is associated with the activation of the cGAS-STING pathway. As one of the hallmarks of malignant tumors, genomic instability plays an important role in the occurrence and development of tumors 40,41. Genomic instability could facilitate the evolution of tumors. However, as the old saying goes, “every coin has two sides”: genomic instability also makes tumor cells bear a higher neoantigen load, which is more easily recognized by the immune system 42,43. The activation of the anti-tumor immune response requires the participation of the innate immune response. The instability of the tumor genome also causes the up-regulation of the cGAS-STING pathway 44,45, which in turn activates the innate immune response (Figure 8).

By analyzing the expression profile of patients with different HRD scores, we identified that *CXCL1*,* CXCL10*,* CXCL11*, *CCL8*, *CCL13* and *CCL18* expression were enriched in patients with high HRD scores, and that the expression of *CXCL10* had the strongest ability to predict the prognosis of the patients. Although recent studies have found that in different types of cancer, patients with high expression of *CXCL10* have a better clinical prognosis 46-48, but no one has explained the molecular mechanism of *CXCL10* high expression. Our cohort analysis combined with the single-cell RNA-seq results fully demonstrated the correlation between HRD and* CXCL10*. As a downstream target gene of the cGAS-STING pathway 49,50, *CXCL10* is a small cytokine belonging to the CXC chemokine family that is also known as Interferon-inducible T-cell alpha chemoattractant and Interferon-gamma-inducible proteins 51,52. *CXCL10* has been attributed to several roles, such as chemoattraction for monocytes/macrophages, T cells, NK cells, dendritic cells, and promotion of T cell adhesion to endothelial cells 53-56. Our results demonstrated that the correlation between genomic instability and activated the cGAS-STING signaling in dMMR tumors 7 can be extended to HRD tumors. Most importantly, we introduced *CXCL10* as a potentially reliable biomarker for the efficacy of ICB therapy. The *CXCL10* can be used as a barometer of the HRD score with strong predictive power.

Under this situation, the determination of whether upregulated *CXCL10* unavoidably results from cGAS-STING activation needs further experimental testing. As a downstream target of the cCAS-STING pathway, upregulated *CXCL10* showed superior predictive power compared with the HRD score. Importantly, the clinical examination of *CXCL10* in tumor tissues or serum is more feasible than applying the steps necessary for calculating the HRD score, and the prediction accuracy of upregulated *CXCL10* is even better than the HRD score itself. We introduced, for the first time, a prospective biomarker associated with the efficacy of immunotherapy in HRD tumors, which merits further investigation in multiple cohorts.

## Supplementary Material

Supplementary table S1.

Supplementary table S2.

Supplementary table S3.

## Abbreviations

HR: homologous recombination
HRD: homologous recombination deficiency
PAPRi: PAPR inhibitor
TIME: tumor immune microenvironment
ICB: immune checkpoint blockade
OSC: ovarian serous cystadenocarcinoma
TNBC: triple-negative breast cancer
HR: homologous recombination repair
TCGA: the Cancer Genome Atlas
GEO: Gene Expression Omnibus
*CXCL10*: C-X-C motif chemokine 10
TPM: transcripts per million
CNV: copy number variation
LOH: loss of heterozygosity
LST: Large-scale State Transitions
TAI: Telomeric Allelic Imbalance
GSEA: Gene set enrichment analysis
DEGs: differentially expressed genes
MSI: microsatellite instability
MAD: median absolute deviation
